# Pregnancy and infection: using disease pathogenesis to inform vaccine strategy

**DOI:** 10.1038/s41541-017-0042-4

**Published:** 2018-02-01

**Authors:** Meghan S. Vermillion, Sabra L. Klein

**Affiliations:** 1 W. Harry Feinstone Department of Molecular Microbiology and Immunology, The Johns Hopkins Bloomberg School of Public Health, Baltimore, MD 21205 USA; 2 Department of Molecular and Comparative Pathobiology, The Johns Hopkins School of Medicine, Baltimore, MD 21205 USA

## Abstract

Vaccination is the mainstay of preventative medicine for many infectious diseases. Pregnant women, unborn fetuses, and neonates represent three at-risk populations that can be simultaneously protected by strategic vaccination protocols. Because the pathogenesis of different infectious microbes varies based on tissue tropism, timing of infection, and host susceptibility, the goals of immunization are not uniform across all vaccines. Mechanistic understanding of infectious disease pathogenesis and immune responses is therefore essential to inform vaccine design and the implementation of appropriate immunization protocols that optimize protection of pregnant women, fetuses, and neonates.

## Introduction

**Fig. 1:**
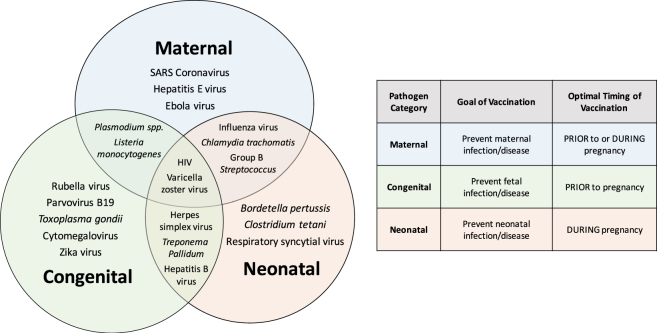
Infectious microbes that cause maternal, congenital, or postnatal complications. The infectious microbes are categorized according to the mechanism of transmission and disease, and the population at greatest risk for severe outcome during or after pregnancy. Infection with some pathogens (e.g., SARS coronavirus, hepatitis E virus, and Ebola virus) during pregnancy cause severe disease in pregnant women, but are not transmitted to offspring. Other infectious microbes (e.g., *Toxoplasma gondii*, rubella virus, parvovirus B19, cytomegalovirus, and Zika viruses) infect and cause mild or asymptomatic disease in pregnant females, but can be vertically transmitted to the fetus and congenital complications. Another category of microbes (e.g., *Bordetella pertussis, Clostridium tetani*, and respiratory syncytial virus) pose the largest risk to neonates after birth. Many infectious microbes (e.g., *Listeria monocytogenes, Plasmodium spp*., HIV, VZV, influenza viruses, *Chlamydia trachomatis*, GBS, *Treponema pallidum*, and herpes viruses) may cause overlapping syndromes depending on the timing of infection during pregnancy. Understanding the pathogenesis of infectious diseases during pregnancy should inform vaccine design and the implementation of appropriate immunization protocols that optimize protection of pregnant women, fetuses and neonates

Vaccination significantly reduces the health burden of many infectious disease, especially in high-risk populations. Pregnant women, unborn fetuses, and neonates represent three populations of high-risk individuals that can all be simultaneously protected from vaccine-preventable infectious disease with strategic maternal immunization protocols. Infectious microbes that pose significant health risks during pregnancy can be divided into three broad categories, based on the pathogenesis and disease outcome (Fig. 1), with some microbes falling within more than one category. First are maternal infections, which are defined by heightened disease severity in pregnant females, but with rare or inconsequential transmission and disease in the fetus. Second are fetal or congenital infections, which are characterized by mild or no disease in pregnant females, but occasional vertical transmission and severe congenital disease in the fetus. Third are neonatal and infant infections, which are not considered to pose significant risk to pregnant women or unborn fetuses, but can cause severe, and sometimes fatal disease in neonates and infants that lack protective maternal immunity following birth.

The vaccination strategies employed differ for micobes within each of these categories and vary based on the at-risk individual (i.e., mother, fetus, and/or neonate/infant), the timing of the greatest risk of infection (i.e., early pregnancy, late pregnancy, or post-natal), and on the duration of protective immunity following vaccination. In this review, we discuss evidence to suggest that immunization strategies for pregnant women should be tailored to optimize protection for the mother, fetus, neonate, infant, or all individuals. We review vaccine-preventable infections during pregnancy and the current vaccination strategies employed to reduce the burden of infectious diseases, including influenza. Further, we examine novel vaccine platforms and consider how their application may provide safe alternatives for enhancing protection of pregnant women. Finally, we discuss vaccine development and prevention strategies for combatting emerging infectious diseases, including Zika, that pose a threat to pregnant women and their fetuses.

## Vaccination against maternal infections

Owing to physiologic and immunologic changes that support pregnancy and tolerance of a semi-allogenic fetus,^1^ pregnant women demonstrate increased susceptibility to certain infectious agents including hepatitis E, varicella zoster, and influenza viruses. Infection with these viruses during pregnancy results in severe maternal disease, increased maternal mortality and associated pregnancy complications, which are observed most frequently during the third trimester and peripartum period. For example, the case fatality rate among pregnant women infected with hepatitis E virus is estimated to be 5–25%,^2,3^ compared with 1–3% in the general population.^4^ Approximately 28% of cases of varicella pneumonia in adults reported from 1965–1989 were from pregnant women^5^; and pregnant women infected with the pandemic 2009 H1N1 influenza A virus (IAV) were reportedly 4 times more likely to be hospitalized or die than the general population.^6^ Overall, vertical transmission of these viruses is relatively uncommon, but adverse pregnancy outcomes, including spontaneous abortion and pre-term birth can still occur as an indirect consequence of maternal inflammation.^7,8^ Reports during the 2009 H1N1 pandemic in Australia and New Zealand indicated that among pregnant women who were hospitalized with suspected H1N1 IAV infection, 50% of their infants required intensive care, and 10% were either stillborn or died shortly after birth; only 2 infants, however, had detectable 2009 H1N1 infection.^9^

The primary goal of vaccination strategies for protecting against maternal infections is the generation of protective maternal immunity either prior to or during early pregnancy. Optimally, vaccination should prevent or reduce disease by inducing sterilizing immunity (i.e., immunity that completely prevents infection). Despite reported reductions in antiviral proteins during pregnancy,^10^ studies comparing vaccine responses between pregnant and nonpregnant women find no difference in either the magnitude or duration of antibody responses against influenza A viruses.^11–13^ In fact, surveillance data from Taiwan reveal that influenza vaccination during pregnancy results in higher levels of seroprotection than does vaccination prior to conception,^14^ with no effect of gestational age on vaccine-induced antibody responses.^15^ As of 2004, the Centers for Disease Control (CDC) Advisory Committee on Immunization Practices (ACIP) categorizes pregnant women as a target population for receiving the inactivated influenza vaccine and recommends that pregnant women be immunized during any trimester.^16^ Several studies evaluating adverse vaccine reactions in pregnant women have concluded that there is no link between pregnancy complications or adverse fetal outcomes among women who are vaccinated during pregnancy.^17–20^ Although the live attenuated intranasal influenza vaccine is not recommended for pregnant women, accidental administration during pregnancy was not associated with an increased risk of adverse reactions.^17,21^ Despite the plethora of data that support the benefits and safety of influenza vaccination during pregnancy, coverage remains low, with a less than 50% maternal vaccination rate during the 2010–2011 influenza season in the United States.^22^ Misconceptions about the safety and benefits of influenza vaccination represent the largest barriers to vaccine acceptance among pregnant women.^23^

Varicella zoster virus (VZV) is another vaccine-preventable infection associated with increased severity during pregnancy.^24^ VZV is an alpha herpes virus and the causative agent of varicella or chickenpox. In temperate climates, seroprevalence among individuals over 30 years of age is estimated to be 95%, with almost 90% of infections occurring prior to 15 years of age.^25^ The first modified-live vaccine against varicella zoster virus was licensed in the United States in 1995, and is now recommended for children over 12 months of age.^26^ Primary VZV infection during pregnancy is therefore uncommon, as most women of childbearing age have been either infected or immunized. In women who have not been previously exposed, however, primary VZV infection between weeks 8 through 26 of gestation is associated with a 2% risk of congenital transmission and disease in the offspring.^27^ Because all licensed VZV vaccines contain live-attenuated virus, their use during pregnancy is contraindicated. Instead, the CDC recommends that nonpregnant women of childbearing age be vaccinated against VZV at least one month prior to conception.^26^ As a herpes virus, infection with VZV is life-long, and reactivation occurs in approximately 10–30% of individuals, which results in a painful skin condition known as shingles or herpes zoster.^28^ Reactivated VZV, however, is not associated with increased disease severity or congenital infection during pregnancy.^24^

Acute viral hepatitis caused by hepatitis E virus (HEV) is an emerging infectious disease that causes severe disease in pregnant women, with a fatality rate of up to 30% in endemic regions.^29^ In addition to heightened maternal disease severity, HEV infection during pregnancy is associated with increased rates of premature birth and prenatal mortality.^30^ Although vertical HEV transmission rates are high, with estimates between 23–50%,^31^ the relative contributions of fetal HEV infection to adverse perinatal outcomes is unclear.^32^ A recombinant HEV subunit vaccine has been developed and proven safe and effective following completion of Phase II and III clinical trials,^33^ but commercial use is currently limited to China. Furthermore, the vaccine is not approved for use in pregnant women despite being 100% efficacious in participants receiving all three doses.^34^ Additional HEV vaccine candidates are being tested in preclinical pregnant animal models, and one recombinant HEV vaccine has been shown to be safe and highly immunogenic in pregnant mice.^35^ Additional studies in a susceptible animal model are needed to confirm efficacy following virus challenge.

## Vaccination against congenital infections

Developing fetuses are extremely vulnerable to both infectious and noninfectious insults. Certain infectious agents that are often clinically silent in healthy adults can cause severe birth defects if they breach the placental barrier during critical developmental periods during pregnancy. An increasing number of pathogens are being recognized for causing congenital disease, and what was originally designated as the TORCH complex (*Toxoplasma gondii*, “other,” Rubella virus, Cytomegalovirus, and Herpes Simplex virus) is now expanded to include other infectious agents including Zika virus. Development of congenital disease can depend on the timing of infection during gestation, the infectious burden, and the pathogenesis in the fetus. The congenital syndrome for each pathogen is characterized by a variety of different developmental abnormalities, and commonly impact hearing, vision, and central nervous system function.^36^ For many congenital infections, the timing of infection during gestation determines the relative risk to the fetus and dictates the spectrum of disease that results. For example, while infection with rubella virus during the first 9 weeks of gestation is associated with an 85% risk of congenital rubella syndrome (CRS), this is reduced to a 52% risk between 9–12 weeks, and minimal risk for infections occurring after 16 weeks of gestation.^37^ In contrast, the risk of congenital toxoplasmosis has been demonstrated to be highest during third trimester pregnancy, which is hypothesized to be due to differential expression of placental toll-like receptors, including TLR6, within first compared with third trimester trophoblast cells.^38^ Similarly, maternal infection with *Listeria monocytogenes* is typically associated with adverse pregnancy outcomes during the third trimester,^39^ though infection during the first trimester in nonhuman primates also leads to rapid fetal demise.^40^

The primary goal of vaccination strategies for protecting against *fetal infections* is generation of protective maternal immunity prior to pregnancy. Because congenital infections can occur in the absence of maternal symptoms, vaccines against congenital agents should ideally provide complete sterilizing immunity. Rubella is included in a live-attenuated combination vaccine for measles, mumps, and rubella (MMR), which confers lifelong protective immunity.^41^ Because of the long duration of protective immunity following rubella vaccination, target populations include children and adolescent girls. However, incomplete vaccination coverage can lead to paradoxical increases in CRS due to an increase in the average age of infection,^42^ and so it is also recommended that unvaccinated women of childbearing age be counseled to receive the rubella vaccine at least one month prior to conception.^43^ The implementation of large-scale rubella vaccination programs has resulted in sufficient population-level immunity, significant reductions in CRS,^44^ and elimination of rubella virus from several developed countries, including the United States.^45^

Following successful implementation of MMR vaccination programs, cytomegalovirus (CMV) has emerged as the most common congenital viral infection in the developed world.^46^ The incidence of congenital CMV varies based on geographic region and socioeconomic status, but overall birth prevalence is estimated to be 0.64%, which is similar to the incidence of Down syndrome and fetal alcohol syndrome.^47^ In contrast to rubella virus, however, there is currently no licensed vaccine available for CMV, and with seroprevalence approaching 100% in some developing countries,^48^ vaccine development has been identified as a priority public healthcare goal.^49,50^ While CMV infection of healthy adults is usually asymptomatic, adaptive immune responses are insufficient to clear the infection, which results in lifelong latent infection of myeloid precursor cells.^51^ Although latent or reactivated CMV is less likely to cause congenital infection than a primary CMV infection during pregnancy,^52,53^ preconception immunity does not completely eliminate transplacental transmission and congenital disease. Moreover, pregnant women with latent CMV infection are still susceptible to primary infection with different CMV strains, which have been shown to have distinct virulence patterns.^54,55^ Overcoming the challenges associated with latent infections and strain variability are significant hurdles in the development of an effective CMV vaccine, and despite significant advances in our knowledge of CMV pathogenesis, the precise immune targets that constitute fetal protection remain unknown. Of the several CMV vaccine candidates that have been tested, none have provided complete protection against infection, and all have failed to protect against reactivation of latent CMV.^56^ More research on the pathogenesis CMV infection is needed to define immunological correlates of protection against CMV transmission during pregnancy to inform vaccine development.

Although not associated with congenital disease, hepatitis B virus (HBV) is another vaccine-preventable infection that can cross the placenta during pregnancy. Mother-to-child-transmission remains the most common route of infection in endemic regions,^57^ and women with active viral replication have up to a 90% chance of vertical transmission.^58^ Of those that are infected perinatally, up to 90% develop chronic HBV infection.^57^ Since the initial recommendation of routine HBV vaccination of children in 1991, the rate of new HBV infections has significantly declined in the United States, but chronic HBV remains prevalent in sub-Saharan Africa and East Asia. Although combined passive and active immunoprophylaxis of infants has significantly reduced perinatal HBV infection, perinatal transmission occurs in up to 20% of infected mothers.^59^ To augment neonatal prophylactic strategies, the CDC ACIP recommends that pregnant women who are identified as being at risk for HBV infection be vaccinated with the recombinant HBV vaccine.^60^ Immunity following receipt of the HBV vaccine is long-lived, with anti-HBV antibodies persisting in most adults for at least 20 years.^61^ Because of the long-term protection conferred by the HBV vaccine, immunization is not necessary for pregnant women who have already been vaccinated and are at low risk of infection.^60^

## Vaccination against neonatal and infant infections

Owing to the limited exposure to foreign antigen and blunted innate immune responses in utero, the neonatal immune system is immature at birth, making neonates (i.e., less than one month of age) particularly susceptible infections.^62^ Infectious diseases are responsible for over 60% of child mortality, and over 40% of these deaths occur within one month of age.^63^ During the neonatal period of immune system maturation, protection against pathogens relies primarily on passive immunity from maternal-derived IgG antibodies. In humans, most maternal antibodies are transferred into the fetal circulation through the placenta prior to birth, which contrasts with most veterinary species, in which maternal antibody is transferred via colostrum immediately following birth. Regardless of species, vaccination during pregnancy increases circulating maternal antibodies and enhances transfer to the fetus/neonate.^14^

The goal of vaccination strategies for protecting against neonatal infections is generation of robust maternal antibody responses during pregnancy to enhance placental transfer. Further, because neonatal protection is exclusively conferred by maternal-derived antibody, vaccines aimed at protecting infants should prioritize induction of humoral over cellular immune responses, with the induction of IgG1 being most important because this IgG isotype is associated with the highest placental transport efficiency in females.^64^ Moreover, the kinetics of maternal vaccine-induced antibody response, the efficiency of placental antibody transfer, and the half-life of the antibody in the neonate should inform the optimal timing of vaccination during pregnancy. Because the peak antibody response is typically observed 1–3 weeks following immunization, vaccination during pregnancy as opposed to before conception is likely to result in the greatest benefit to the neonate. Further, the efficiency of placental antibody transfer in females increases throughout gestation, with less than 8% maternal IgG transferred to the fetus in the first 16 weeks of gestation,^65^ significantly more transferred during the second and third trimesters, and at delivery fetal IgG often exceeds maternal levels.^66^ Vaccination of females during the second and third trimesters of pregnancy is most likely to generate the greatest level of protection in the neonate, but the precise timing for maximum protection is debated. Controversy over the timing of pertussis vaccination in pregnancy has been reviewed elsewhere,^67^ with some reports claiming peak cord blood antibody concentrations following vaccination in the second trimester, and others reporting peak antibody concentrations following vaccination in the third trimester. Antibody avidity also influences the efficiency of placental transfer, with higher avidity antibodies crossing the placenta with greater efficiently than low avidity antibodies.^68^ More consideration should be given to the development of high avidity antibodies in the timing of vaccination during pregnancy, as protective immunity in the infant depends on both the concentration and avidity of the maternal-derived antibody.

The half-life of maternal antibodies in infants also must be considered in vaccine development and administration. Maternal-derived IgG1 is reported to have a half-life of approximately 48 days in serum,^69^ and depending on serum antibody titers present at birth, this translates into protective immunity for approximately the first 3–9 months of life for most infant pathogens.^70^ The half-life of the antibody also dictates the vaccination schedules for infants, as the presence of maternal-derived antibody interferes with vaccine efficacy, and it is not until maternal-derived antibody has waned below a certain threshold that an infant can mount its own active vaccine response. The goal of the infant vaccine series is to time vaccination to coincide with the time that maternal-derived antibody drops below the threshold at which it can neutralize the vaccine antigen. Because the precise timing of these events is unpredictable, infant vaccination schedules are designed so that vaccines are administered in a series that spans the duration of this window, and minimize susceptibility to natural infection. In the United States, infant vaccines are recommended at 2, 4 and 6 months of age.^41^

*Bordetella pertussis* is a vaccine-preventable respiratory pathogen of significant public health importance, and it is a major cause of mortality in infants lacking protective maternal immunity. Vaccination of women during pregnancy, however, significantly enhances the transfer of maternal antibody to the fetus,^71,72^ and these newborns are 11 times more likely to have protective antibody titers at birth compared with those born from women who were not vaccinated during pregnancy.^71^ Inactivated pertussis antigen is combined with tetanus and diphtheria toxoids in a single vaccine (Tdap), which the CDC ACIP recommends for all pregnant women, regardless of previous vaccine history.^73^ In contrast to vaccine formulations that contain killed whole *B. pertussis* organisms, the Tdap vaccine contains only select antigens and confers relatively weak and only transient protective immunity that declines after 1 year.^74^ Vaccination of women either prior to conception or during early pregnancy does not provide adequate neonatal protection against pertussis.^75^ Consequently, the CDC considers the third trimester to be the optimal time to administer the Tdap vaccine to pregnant women.^73^ Adverse events reported following Tdap vaccination are generally mild, and there are no reported risks of adverse pregnancy outcomes related to Tdap vaccination during pregnancy.^76^ Despite consistent evidence that supports the benefit and safety of Tdap vaccination during pregnancy, coverage remains low, with an estimated 42% of pregnant women receiving the Tdap vaccine in the United States in 2013.^77^

Receipt of the Tdap vaccine during pregnancy also confers protection against neonatal tetanus, which is associated with case fatality approaching 100% in the absence of medical care.^78^ Disease is caused by the toxin produced by *Clostridium tetani*, and infection occurs most commonly due to contamination of the umbilical stump following delivery. Consequently, the incidence of disease is much greater in developing countries, where maternal vaccination is scarce and perinatal hygiene practices are poor.^79^ In 1989, the World Health Assembly called for the elimination of neonatal tetanus, which has inspired an initiative to improve vaccination coverage and birth hygiene in 59 countries with high disease prevalence. As part of this initiative, immunization standards have been expanded and recommend that pregnant women with unknown or inadequate vaccination history receive two doses of the toxoid-containing vaccine, administered one month apart.^80^ Maternal anti-tetanus antibodies are passively transferred to the fetus, and it is estimated that maternal immunization reduces neonatal tetanus mortality by 94%.^81^

Respiratory syncytial virus (RSV) is the most common respiratory viral pathogen of newborns and infants, and accounts for 50–90% of acute bronchiolitis and 5–20% of pneumonia cases in hospitalized children less than 2 years of age.^82^ RSV is also reported to cause severe disease and hospitalization in pregnant women when infection occurs during the third trimester,^83,84^ and therefore dually qualifies as a maternal infection as well. A licensed vaccine against RSV is currently unavailable, but several vaccine candidates have shown promise in various animal models.^85–88^ Given the importance of this pathogen during early life, vaccine development strategies have focused on maternal immunization, with three maternal vaccines currently in clinical trials.^89^ Maternal vaccination against RSV has direct and indirect benefits to the neonate; neonates are directly protected through passive transfer of maternal antibody through the placenta, and they are indirectly protected because a vaccinated mother is less likely to transmit the infection to her infant.^90^

## Contributions of pregnant animal models

Vaccination of pregnant women is controversial, and immunization with live (i.e., replication-competent) viral or bacterial vaccines is generally contraindicated due to the theoretical risk of congenital infection and teratogenic effects from the vaccine strains. However, in a report of over 2000 pregnant women who were unknowingly immunized with live attenuated rubella vaccine, there were no cases of vaccine-associated congenital rubella infection,^91^ and live virus strains of influenza or yellow fever viruses administered to pregnant women also have no link with pregnancy complications.^21,92^ Vaccination with inactivated vaccines such as influenza and Tdap during pregnancy have low uptake, with concerns of safety among both patients and their healthcare providers being a primary barrier. The safety of vaccine adjuvants is debated, and although neither the Tdap nor seasonal influenza vaccine recommended during pregnancy contain adjuvants, retrospective studies evaluating safety of the adjuvanted pandemic H1N1 influenza vaccine in pregnant women found no relationship with adverse pregnancy outcomes.^93^ The conservative approach to vaccination protocols for pregnant women stems from the lack of controlled safety and efficacy studies for this population. For ethical reasons, pregnant women are exempted from almost all clinical and vaccine trials, and heath care providers are less likely to endorse prophylactic treatments for which safety and efficacy profiles have not been adequately characterized.

Whereas study in pregnant women is not possible, pre-clinical testing in animal models may provide a useful alternative, and vaccine preclinical trials in pregnant animal models may provide information to inform healthcare policies for pregnant women. Although there are some differences in the length of gestation, placental structure, and fetal development between humans and animal models, many structural and functional parallels exist,^94–96^ which serve as tractable platforms for evaluating the safety and efficacy of various therapies during pregnancy.

Similar to humans, pregnant mice, rats, and rabbits have a hemochorial placenta, and their relatively short gestation and large litters are advantageous for performing high throughput screening of candidate therapeutics for safety and efficacy. Preclinical behavioral testing of rodent offspring has proven to be a promising avenue for identifying and predicting adverse effects associated with prenatal drug exposure in children.^97^ Both rodent and rabbit models have been instrumental in testing teratogenic effects of artemisinin-based combination therapies for treating malaria in pregnant women. These studies concluded that drug-related teratogenic effects are limited to the first trimester, which supports the World Health Organization (WHO) recommendation that artemisinin may be administered only during the second or third trimester in pregnant women.^98,99^ One limitation of mouse and rat models, however, is their inability to recapitulate certain elements of human congenital disease. For instance, because murine CMV is not transmitted vertically as it is in humans, other animal models, including guinea pigs and nonhuman primates, are required for studying this aspect of disease pathogenesis. Studies in pregnant nonhuman primates have been instrumental for the identification of CD4 + T cell responses as critical for early control of CMV infection and transmission during pregnancy,^100^ and studies in guinea pigs have demonstrated that a single-cycle infectious CMV vaccine induces immune responses similar to natural infection and protects against congenital infection.^101^ Guinea pigs are also a useful model of chlamydial genital infection in humans. Experimental venereal infection with *Chlamydophila caviae* mimics disease associated with *C. trachomatis* in humans, including both sexual and perinatal transmission. Guinea pigs have therefore served as a useful model for testing candidate vaccines and treatments.^102^

Rabbits continue to serve as an important model of venereal infection with *Treponema pallidum*, the causative agent of syphilis, which is associated with congenital disease in humans. While natural infection in rabbits is associated with the species-specific *T. paraluiscanuculi*, rabbits can be experimentally inoculated with human *T. pallidum*, and have been instrumental in testing the efficacy of candidate vaccines.^103^ Many mammalian species, including rodents,^104^ ruminants,^105^ and nonhuman primates,^40,106^ are susceptible to infection with *Listeria monocytogenes* and demonstrate similar fetal complications when infection occurs during pregnancy. Studies in various animal models have uniquely contributed to our understanding of placental listeriosis and serve as a platform for evaluating prevention strategies.^107,108^ Finally, mice,^85^ cotton rats,^86^ guinea pigs,^88^ and sheep^87^ are all susceptible to infection with RSV, and vaccination of pregnant animals has facilitated the development and testing of maternal immunization strategies for protecting against neonatal RSV. Based on preliminary studies in guinea pigs,^88^ an experimental RSV recombinant F nanoparticle vaccine is now being evaluated in third-trimester pregnant women (Clintrials.gov, NCT02247726).

Beyond the direct modeling of human congenital infection in animals, information can also be gained from the study of related veterinary pathogens. For example, bovine viral diarrhea virus (BVDV) is an important reproductive pathogen that infects cattle worldwide, and infection during pregnancy causes congenital infection and disease. Persistently infected animals serve as reservoirs within a herd and can have a huge agricultural financial impact.^109^ As a result, significant resources have been dedicated to the development and optimization of BVDV vaccines and vaccine protocols, considering variables such as the type and timing of vaccination on immune response and protection against challenge.^110^ The information gleaned from these studies may inform vaccine development and optimization protocols for related pathogens in pregnant women, for which similar studies cannot ethically be performed.

## Vaccine strategies for emerging infectious diseases

Zika virus (ZIKV) is a unique Flavivirus that causes mild or subclinical disease in pregnant women,^111,112^ but can have devastating effects for the fetus and neonate. Infection during pregnancy is linked with spontaneous abortion and a variety of birth defects, including microcephaly and impaired neurocognitive function.^113^ Since its initial discovery in African macaques in 1947, ZIKV has expanded its geographical range and evolved into separate virus lineages, with environmental pressures resulting in the emergence of virulent substrains, raising concerns about vaccine escape mutants once a vaccine is approved.^114^ The combination of its unique pathogenesis, diverse modes of transmission, and rapid global spread has increased efforts toward development and licensing of a ZIKV vaccine.

A basic understanding of ZIKV biology and pathogenesis is essential for development of an effective vaccine against ZIKV. Although we can extrapolate some biological information from related *Flaviviruses*, such as yellow fever, West Nile, and dengue viruses, ZIKV has unique characteristics following in vivo infection, which pose significant challenges to vaccine design. Unlike other *Flaviviruses*, ZIKV has a tropism for reproductive tissues, including the testes, semen, and sperm in males^115,116^ and the placenta in pregnant females,^117–119^ which is hypothesized to contribute to sexual and vertical modes of transmission, respectively. In addition to unique tissue tropisms, ZIKV persists in reproductive tissue following clearance of systemic viremia. In males, ZIKV RNA can be detected for months following recovery from symptomatic infection,^120,121^ and virus persistence in the placenta of pregnant women is hypothesized to contribute to prolonged viremia in this population.^122^ Evidence of virus persistence suggests that ZIKV may have evolved mechanisms for evasion of host immune responses when infection occurs in certain immune-privileged tissues. Scenarios involving persistent ZIKV infections should be considered in developing and testing candidate vaccines. Considering the potential for viral persistence in the semen, vaccinating men may serve as an additional strategy to reduce transmission to the fetus, as pregnant women may be infected by their sex partners.

Characterization of the immune response to ZIKV infection is essential for determining correlates of protection for vaccine efficacy. Following infection of both humans and nonhuman animals, ZIKV induces neutralizing antibodies against the ZIKV E protein, which prevent fetal infection and demise when administered to pregnant mice.^123^ Further, 26 MHC Class I epitopes have been identified that conferred protection against ZIKV challenge in immunocompetent mice.^124^ To date, 45 candidate ZIKV vaccines have been developed, and as of February 2017, nine have entered Phase I clinical trials.^125^ Vaccine candidates have been developed using diverse platforms, including DNA, mRNA, and purified inactivated and live-attenuated virus, many of which have been tested in non-pregnant mouse and nonhuman primate models for their ability to generate immune responses that mimic responses to natural infection and protected against ZIKV challenge. A candidate DNA plasmid vaccine induced robust cellular immunity and neutralizing antibody responses in both nonhuman primates and immunocompetent mice, and conferred complete protection against lethal ZIKV challenge in type I interferon receptor deficient (IFNAR −/−) mice.^126^ LNP-mRNA vaccines induced similar protective immunity, which was characterized by high neutralizing antibody titers and sterilizing immunity against ZIKV challenge in non-pregnant mice and nonhuman primates.^127,128^ Whether these candidate vaccines induce protective immunity in pregnant females that is sufficient to prevent fetal and neonatal infections^117^ requires further evaluation. Also, whether pre-existing immunity to other *Flaviviruses* that co-circulate with ZIKV, including dengue virus and West Nile virus, affects the efficacy of ZIKV vaccines in pregnant females should be considered in both preclinical animal models and human clinical trials.

## Application of novel vaccine platforms

Conventional vaccines are formulated from either live, attenuated pathogen strains or from inactivated pathogens, but there are notable disadvantages to each of these platforms. Live, attenuated vaccines are replication-competent with the potential of becoming virulent and causing adverse effects in individuals with weakened immune systems. Due to the unknown risk to the developing fetus, live virus vaccines are not recommended for use in pregnant women. Inactivated vaccines, on the other hand, are not associated with a risk of reacquisition of virulence, but they tend to induce a weaker host immune response.^129^ Efforts to balance safety with immunogenicity have led to the development of several novel vaccine technologies, including replication-deficient nanoparticle-based vaccines and self-assembling recombinant virus-like particles (VLPs), replication-competent recombinant viral vectors, and single-cycle infectious viruses that can infect, but not replicate in host cells.

Nanoparticle delivery platforms, including liposomes and synthetic polymers, can be engineered to enhance selective tissue homing for high potency, targeted delivery of antigen in its native conformation.^130^ Recombinant VLPs combine highly immunogenic surface viral proteins with encapsulated adjuvants that are devoid of infectious nucleic acid, but induce strong cellular and humoral immune responses.^131^ Both nanoparticle and VLP vaccines are devoid of genetic material and are therefore replication incompetent, enhancing safety in vulnerable individuals. Although current production yields and costs associated with these technologies may prohibit large-scale use, these platforms may be well-suited for targeted use in high-risk populations, including pregnant women. Since the first nanoparticle-based vaccine was licensed for hepatitis B virus in 1981, the technology has been applied to develop licensed vaccines against human papilloma virus, hepatitis E virus, and malaria.^131^ Demonstration of safety and efficacy during pregnancy has not yet been documented.

Recombinant viruses can be engineered to combine the antigenic genes of one virus with the structural genes of another. This targeted manipulation of the virus genome is used to remove virulence genes to enhance safety and alter envelope proteins to change cell tropism. Recombinant viruses can be engineered to retain the ability to infect and replicate in the host, while preserving infectious potential and enhancing the generation of innate and adaptive immune responses that mimic natural infection. Recombinant virus vaccines, however, warrant careful consideration of the safety of the vector itself, especially in pregnant women. Once the safety and efficacy profiles of a viral vector platform have been established, engineering new antigenic targets into the viral genome are relatively simple, and do not require extensive re-validation, as safety and efficacy is most influenced by the vector virus. This can significantly reduce the time to develop and manufacture vaccines against new viral pathogens.^132^ Viruses from many different families can be used as vectors provided they can infect the host and elicit a productive immune response without causing disease. Of note, poxviruses are practical vectors due to ease of growth and manipulation in vitro, wide host range, and robust induction of protective immune responses. Although vaccinia virus vectors are contraindicated during pregnancy due to risk of disseminated disease, recent testing of a raccoonpoxvirus-vectored rabies vaccine in pregnant mice proved safe and effective.^133^

Other novel vaccine platforms are based on genetic engineering and creation of targeted loss-of-function mutations in the viral genome. Reverse genetics technology has contributed the identification of the sequences within the viral genomes that are essential for infection and replication *in vivo*. It is now possible to engineer recombinant virus vaccines with targeted mutations in a cost- and time-efficient manner. In contrast to inactivated vaccines, reverse genetics allows for controlled manipulation of the viral genome that targets a specific process in the virus life cycle. This enables production of a vaccine strain with maximum efficacy and safety, and represents another promising alternative to conventional attenuated or killed virus vaccines. Among the safest recombinant vaccine approaches are the single-cycle infectious (sci) viruses, which have been developed from influenza A virus (IAV) backbones by deleting or truncating viral proteins necessary for completion of the virus life cycle within the host.^134^ Such genetic modifications render the virus replication-incompetent, but capable of infecting and inducing an immune response in the host.^135^ The infectious capacity of sciIAV vaccines results in strong cellular and humoral immune responses, without the risks and adverse effects associated with live-attenuated vaccine strains. Studies in nonpregnant mice have demonstrated that a single dose of sciIAV vaccine confers protection against heterosubtypic lethal challenge without any adverse effects.^136–141^ Similar safety and efficacy profiles of sciIAV have been replicated in ferret and pig models,^136,142^ but studies in pregnant animals have not been performed. Compared with the risks associated with live virus vaccination and concerns of efficacy with inactivated vaccines, novel vaccine platforms, such as nanoparticle-based technologies, VLPs and replication-deficient viruses have proven benefits.^130^ Additional safety and efficacy studies in pregnancy models are warranted to validate and expand the use of these vaccine platforms for pregnant women.(Table 1)

**Table 1:** Pathogens that cause adverse pregnancy or perinatal outcomes

Pathogen	Category	Maternal susceptibility and/or disease severity	Vertical transmission and/or disease	Congenital Disease	Neonatal susceptibility and/or disease severity	Prevention	References
*Bordetella pertussis*	bacteria	=	Postpartum	Not documented	↑	Maternal vaccination (Tdap) during second or third trimester pregnancy	(73) (143)
*Chlamydia trachomatis*	bacteria	=	Peripartum	conjunctivitis	↑	antibiotics	(144)
*Clostridium tetani*	bacteria	=	Peripartum	Not documented	↑	Maternal vaccination (Tdap) during second or third trimester pregnancy	(81) (78)
Group B *Streptococcus*	bacteria	=	Peripartum > Transplacental	Preterm birth, vision and hearing loss, mental retardation, cerebral palsy	↑	Intrapartum antibiotic prophylaxis	(145) (146)
*Listeria monocytogenes*	bacteria	↑	Transplacental	Miscarriage, perterm birth, sepsis, meningitis	↑	avoid high risk foods	(147) (39)
*Treponema pallidum*	bacteria	=	Transplacental	Miscarriage, preterm birth, stillbirth, bone deformities, anemia, hepatosplenomegaly, blindness, deafness, meningitis, skin rash		prophylactic antibiotics	(148) (149)
*Plasmodium spp*.	protozoa	↑	Transplacental	Variable		artemisinin-based combination therapies	(150) (151) (152)
*Toxoplasma gondii*	protozoa	**=**	Transplacental	stillbirth, chorioretinitis, deafness, microcephaly, encephalitis, developmental delay		No FDA approved treatment; avoid reservoirs (cat feces, undercooked meat)	(153) (154)
Cytomegalovirus	virus	**=**	Transplacental	Sensorineural hearing loss, mental retardation, cerebral palsy, seizures, chorioretinitis		No vaccine; CMV hyperimmune globulin	(155) (156) (157)
Ebola virus	virus	↑	Transplacental likely	Miscarriage or neonatal death most common	□	Not described	(158) (159) (160)
Hepatitis B virus	virus	**=**	Transplacental or Peripartum	chronic hepatitis	↑	Vaccinate “at-risk” pregnant women	(58) (60)
Hepatitis E virus	virus	↑	Transplacental or Peripartum	influence of transplacental transmission on congenital disease unknown	↑	Vaccination (licensed in China only - not licensed during pregnancy)	(32) (3) (4) (33)
Herpes simplex virus	virus	↑	Peripartum > Transplacental	spontaneous abortion, preterm birth	↑	No vaccine; antivirals not effective	(161) (162)
Human immunodeficiency virus	virus	↑	Transplacental or Peripartum	Not documented	↑	HAART, cesarean delivery, formula feeding	(163) (164)
Influenza virus	virus	↑	Rare	Not documented	↑	Maternal vaccination (TIV) during *any* trimester	(1) (9) (8) (13)
Parvovirus B19	virus	**=**	Transplacental	spontaneous abortion, hydrops fetalis, congenital anomalies, aplastic anemia (rare)		No vaccine; Intrauterine blood transfusion	(165)
Respiratory syncytial virus	virus	= / ↑	Postpartum	Not documented	↑	No vaccine; immunoprophylaxis for at-risk neonates	(84) (85)
Rubella virus	virus	**=**	Transplacental	Sensorineural hearing loss, ocular abnormalities, heart disease, mental retardation		Vaccination (MLV) *prior* to pregnancy	(166) (43)
SARS Coronavirus	virus	↑	Not documented	Not documented		Not described	(167) (168)
Varicella zoster virus	virus	↑	Transplacental or Peripartum	Fetal varicella syndrome (skin scars, ocular defects, limb deformities, prematurity, cortical atrophy)	↑	Vaccination (MLV) *prior* to pregnancy	(5) (169) (26)
Zika virus	virus	**=**	Transplacental	microcephaly, hearing loss, visual impairments, mental retardation, arthrogryposis		No vaccine; Mosquito control, condoms	(170) (117)

Key: →↑ -- susceptibility and/or disease severity is increased compared with the general population

= -- susceptibility and/or disease severity is similar to the general population

## Conclusions

Strategic immunization of women, either prior to or during pregnancy, can eliminate or substantially reduce the risk of maternal, fetal, and neonatal infection and disease. The effectiveness of an immunization protocol depends on both the efficacy of the vaccine in inducing protective immune responses and on the timing of vaccine delivery during pregnancy to synchronize the peak vaccine response with the period of greatest susceptibility in the host. Optimization of vaccination protocols to achieve this goal requires an understanding of the mechanisms of infection and pathogenesis of disease during pregnancy. Successful implementation of vaccine protocols for pregnant women requires consideration of additional challenges, such as the frequency of unplanned pregnancies and access to prenatal health care. Human surveillance data provide correlative clues of the character of specific infections, but mechanistic understanding requires additional study in comparative animal model systems.

## References

[1] Robinson DP, Klein SL (2012). Pregnancy and pregnancy-associated hormones alter immune responses and disease pathogenesis. Horm. Behav..

[2] Khuroo MS, Teli MR, Skidmore S, Sofi MA, Khuroo MI (1981). Incidence and severity of viral hepatitis in pregnancy. Am. J. Med..

[3] Khuroo MS, Kamili S (2003). Aetiology, clinical course and outcome of sporadic acute viral hepatitis in pregnancy. J. Viral Hepat..

[4] Emerson SU, Purcell RH (2003). Hepatitis E virus. Rev. Med. Virol..

[5] Esmonde TF, Herdman G, Anderson G (1989). Chickenpox pneumonia: an association with pregnancy. Thorax.

[6] Jamieson DJ (2009). H1N1 2009 influenza virus infection during pregnancy in the USA. Lancet.

[7] Shi L, Fatemi SH, Sidwell RW, Patterson PH (2003). Maternal influenza infection causes marked behavioral and pharmacological changes in the offspring. J. Neurosci..

[8] Shi L, Tu N, Patterson PH (2005). Maternal influenza infection is likely to alter fetal brain development indirectly: the virus is not detected in the fetus. Int. J. Dev. Neurosci..

[9] Investigators, A. I. & Australasian Maternity Outcomes Surveillance, S. Critical illness due to 2009 A/H1N1 influenza in pregnant and postpartum women: population based cohort study. *BMJ* **340**, c1279 (2010). 10.1136/bmj.c1279 PMC2841744 20299694

[10] Forbes RL, Gibson PG, Murphy VE, Wark PA (2012). Impaired type I and III interferon response to rhinovirus infection during pregnancy and asthma. Thorax.

[11] Hulka JF (1964). Effectiveness of Polyvalent Influenza Vaccine in Pregnancy. Report of a Controlled Study during an Outbreak of Asian Influenza. Obstet. Gynecol..

[12] Murray DL, Imagawa DT, Okada DM, St Geme JW (1979). Antibody response to monovalent A/New Jersey/8/76 influenza vaccine in pregnant women. J. Clin. Microbiol..

[13] Mutsaerts E (2016). Influenza vaccination of pregnant women protects them over two consecutive influenza seasons in a randomized controlled trial. Expert. Rev. Vaccin..

[14] Chao AS (2017). Seropositivity of influenza A H1NI in mothers and infants following maternal vaccination with trivalent seasonal influenza vaccine after the 2009 pandemic. Taiwan. J. Obstet. Gynecol..

[15] Sperling RS (2012). Immunogenicity of trivalent inactivated influenza vaccination received during pregnancy or postpartum. Obstet. Gynecol..

[16] Harper SA (2005). Prevention and control of influenza. Recommendations of the Advisory Committee on Immunization Practices (ACIP). Mmwr. Recomm. Rep..

[17] Moro PL (2011). Adverse events in pregnant women following administration of trivalent inactivated influenza vaccine and live attenuated influenza vaccine in the Vaccine Adverse Event Reporting System, 1990-2009. Am. J. Obstet. Gynecol..

[18] Irving SA (2013). Trivalent inactivated influenza vaccine and spontaneous abortion. Obstet. Gynecol..

[19] Nordin JD (2014). Monovalent H1N1 influenza vaccine safety in pregnant women, risks for acute adverse events. Vaccine.

[20] Kharbanda EO (2013). Inactivated influenza vaccine during pregnancy and risks for adverse obstetric events. Obstet. Gynecol..

[21] Toback SL (2012). Maternal outcomes among pregnant women receiving live attenuated influenza vaccine. Influenza Other Respir. Virus..

[22] Centers for Disease, C. & Prevention. Influenza vaccination coverage among pregnant women --- United States, 2010-11 influenza season. *Mmwr. Morb. Mortal. Wkly. Rep*. **60**, 1078–1082 (2011). 21849964

[23] Meharry PM, Cusson RM, Stiller R, Vazquez M (2014). Maternal influenza vaccination: evaluation of a patient-centered pamphlet designed to increase uptake in pregnancy. Matern. Child. Health J..

[24] Lamont RF (2011). Varicella-zoster virus (chickenpox) infection in pregnancy. BJOG.

[25] Khoshnood B (2006). Seroprevalence of varicella in the French population. Pediatr. Infect. Dis. J..

[26] Prevention of varicella: Recommendations of the Advisory Committee on Immunization Practices (ACIP). Centers for Disease Control and Prevention. *Mmwr. Recomm. Rep*. **45**, 1–36 (1996). 8668119

[27] Gershon AA, Gershon MD (2013). Pathogenesis and current approaches to control of varicella-zoster virus infections. Clin. Microbiol. Rev..

[28] Thomas SL, Hall AJ (2004). What does epidemiology tell us about risk factors for herpes zoster?. Lancet Infect. Dis..

[29] Perez-Gracia, M. T., Suay-Garcia, B. & Mateos-Lindemann, M. L. Hepatitis E and pregnancy: current state. *Rev. Med. Virol.* **27**, e1929 (2017). 10.1002/rmv.1929 28318080

[30] Khuroo MS, Kamili S, Jameel S (1995). Vertical transmission of hepatitis E virus. Lancet.

[31] Kumar RM (2001). Sero-prevalence and mother-to-infant transmission of hepatitis E virus among pregnant women in the United Arab Emirates. Eur. J. Obstet. Gynecol. Reprod. Biol..

[32] Krain LJ, Atwell JE, Nelson KE, Labrique AB (2014). Fetal and neonatal health consequences of vertically transmitted hepatitis E virus infection. Am. J. Trop. Med. Hyg..

[33] Zhu FC (2010). Efficacy and safety of a recombinant hepatitis E vaccine in healthy adults: a large-scale, randomised, double-blind placebo-controlled, phase 3 trial. Lancet.

[34] Li S, Zhang J, Xia N (2015). Lessons from hepatitis E vaccine design. Curr. Opin. Virol..

[35] Joshi SS, Arankalle VA (2015). Enhanced humoral response in pregnant mice immunized with liposome encapsulated recombinant neutralizing epitope protein of Hepatitis- E virus. Virol. J..

[36] Racicot K, Mor G (2017). Risks associated with viral infections during pregnancy. J. Clin. Invest..

[37] Ely JW, Yankowitz J, Bowdler NC (2000). Evaluation of pregnant women exposed to respiratory viruses. Am. Fam. Physician.

[38] Wujcicka W, Wilczynski J, Nowakowska D (2014). Do the placental barrier, parasite genotype and Toll-like receptor polymorphisms contribute to the course of primary infection with various Toxoplasma gondii genotypes in pregnant women?. Eur. J. Clin. Microbiol. Infect. Dis..

[39] Lamont RF (2011). Listeriosis in human pregnancy: a systematic review. J. Perinat. Med..

[40] Wolfe B (2017). Acute Fetal Demise with First Trimester Maternal Infection Resulting from Listeria monocytogenes in a Nonhuman Primate Model. MBio.

[41] National Center for, I. & Respiratory, D. General recommendations on immunization --- recommendations of the Advisory Committee on Immunization Practices (ACIP). *Mmwr. Recomm. Rep*. **60**, 1–64 (2011). 21293327

[42] Metcalf CJ, Lessler J, Klepac P, Cutts F, Grenfell BT (2012). Impact of birth rate, seasonality and transmission rate on minimum levels of coverage needed for rubella vaccination. Epidemiol. Infect..

[43] McLean HQ (2013). Prevention of measles, rubella, congenital rubella syndrome, and mumps, 2013: summary recommendations of the Advisory Committee on Immunization Practices (ACIP). Mmwr. Recomm. Rep..

[44] Khandaker G, Zurynski Y, Jones C (2014). Surveillance for congenital rubella in Australia since 1993: cases reported between 2004 and 2013. Vaccine.

[45] Centers for Disease, C. & Prevention. Elimination of rubella and congenital rubella syndrome--United States, 1969-2004. *Mmwr. Morb. Mortal. Wkly. Rep*. **54**, 279–282 (2005). 15788995

[46] Manicklal S, Emery VC, Lazzarotto T, Boppana SB, Gupta RK (2013). The “silent” global burden of congenital cytomegalovirus. Clin. Microbiol. Rev..

[47] Kenneson A, Cannon MJ (2007). Review and meta-analysis of the epidemiology of congenital cytomegalovirus (CMV) infection. Rev. Med. Virol..

[48] Lanzieri TM (2015). Seroprevalence of cytomegalovirus among children 1 to 5 years of age in the United States from the National Health and Nutrition Examination Survey of 2011 to 2012. Clin. Vaccin. Immunol..

[49] Arvin AM (2004). Vaccine development to prevent cytomegalovirus disease: report from the National Vaccine Advisory Committee. Clin. Infect. Dis..

[50] Griffiths P (2013). Desirability and feasibility of a vaccine against cytomegalovirus. Vaccine.

[51] Sinclair J, Sissons P (2006). Latency and reactivation of human cytomegalovirus. J. Gen. Virol..

[52] Fowler KB, Stagno S, Pass RF (2003). Maternal immunity and prevention of congenital cytomegalovirus infection. JAMA.

[53] Emery VC, Hassan-Walker AF, Burroughs AK, Griffiths PD (2002). Human cytomegalovirus (HCMV) replication dynamics in HCMV-naive and -experienced immunocompromised hosts. J. Infect. Dis..

[54] Arav-Boger R (2015). Strain Variation and Disease Severity in Congenital Cytomegalovirus Infection: In Search of a Viral Marker. Infect. Dis. Clin. North. Am..

[55] Arav-Boger R (2006). Cytomegalovirus (CMV)-encoded UL144 (truncated tumor necrosis factor receptor) and outcome of congenital CMV infection. J. Infect. Dis..

[56] Plachter B (2016). Prospects of a vaccine for the prevention of congenital cytomegalovirus disease. Med. Microbiol. Immunol..

[57] Vodkin I, Patton H (2014). Management of Hepatitis B virus infection during pregnancy. Minerva Gastroenterol. Dietol..

[58] Lee, C., Gong, Y., Brok, J., Boxall, E. H. & Gluud, C. Hepatitis B immunisation for newborn infants of hepatitis B surface antigen-positive mothers. *Cochrane Database Syst Rev* **2**, CD004790 (2006). 10.1002/14651858.CD004790.pub2 PMC13110914 16625613

[59] Yang M, Qin Q, Fang Q, Jiang L, Nie S (2017). Cesarean section to prevent mother-to-child transmission of hepatitis B virus in China: A meta-analysis. Bmc. Pregnancy Childbirth.

[60] Mast EE (2006). A comprehensive immunization strategy to eliminate transmission of hepatitis B virus infection in the United States: recommendations of the Advisory Committee on Immunization Practices (ACIP) Part II: immunization of adults. Mmwr. Recomm. Rep..

[61] Van Damme P, Leroux-Roels G, Suryakiran P, Folschweiller N, Van Der Meeren O (2017). Persistence of antibodies 20 y after vaccination with a combined hepatitis A and B vaccine. Hum. Vaccin Immunother..

[62] Wynn JL, Levy O (2010). Role of innate host defenses in susceptibility to early-onset neonatal sepsis. Clin. Perinatol..

[63] Liu L (2012). Global, regional, and national causes of child mortality: an updated systematic analysis for 2010 with time trends since 2000. Lancet.

[64] Malek A, Sager R, Kuhn P, Nicolaides KH, Schneider H (1996). Evolution of maternofetal transport of immunoglobulins during human pregnancy. Am. J. Reprod. Immunol..

[65] Saji F, Samejima Y, Kamiura S, Koyama M (1999). Dynamics of immunoglobulins at the feto-maternal interface. Rev. Reprod..

[66] Garty BZ, Ludomirsky A, Danon YL, Peter JB, Douglas SD (1994). Placental transfer of immunoglobulin G subclasses. Clin. Diagn. Lab. Immunol..

[67] Calvert A, Jones CE (2017). Placental transfer of antibody and its relationship to vaccination in pregnancy. Curr. Opin. Infect. Dis..

[68] Avanzini MA (1998). Placental transfer favours high avidity IgG antibodies. Acta Paediatr..

[69] Sarvas H, Seppala I, Kurikka S, Siegberg R, Makela O (1993). Half-life of the maternal IgG1 allotype in infants. J. Clin. Immunol..

[70] Nicoara C, Zach K, Trachsel D, Germann D, Matter L (1999). Decay of passively acquired maternal antibodies against measles, mumps, and rubella viruses. Clin. Diagn. Lab. Immunol..

[71] Gall SA, Myers J, Pichichero M (2011). Maternal immunization with tetanus-diphtheria-pertussis vaccine: effect on maternal and neonatal serum antibody levels. Am. J. Obstet. Gynecol..

[72] Leuridan E (2011). Effect of a prepregnancy pertussis booster dose on maternal antibody titers in young infants. Pediatr. Infect. Dis. J..

[73] Centers for Disease, C. & Prevention. Updated recommendations for use of tetanus toxoid, reduced diphtheria toxoid, and acellular pertussis vaccine (Tdap) in pregnant women--Advisory Committee on Immunization Practices (ACIP), 2012. *Mmwr. Morb. Mortal. Wkly. Rep*. **62**, 131–135 (2013). PMC4604886 23425962

[74] Weston WM, Friedland LR, Wu X, Howe B (2011). Immunogenicity and reactogenicity of co-administered tetanus-diphtheria-acellular pertussis (Tdap) and tetravalent meningococcal conjugate (MCV4) vaccines compared to their separate administration. Vaccine.

[75] Healy CM, Rench MA, Baker CJ (2013). Importance of timing of maternal combined tetanus, diphtheria, and acellular pertussis (Tdap) immunization and protection of young infants. Clin. Infect. Dis..

[76] Walls T, Graham P, Petousis-Harris H, Hill L, Austin N (2016). Infant outcomes after exposure to Tdap vaccine in pregnancy: an observational study. BMJ Open..

[77] Barber A, Muscoplat MH, Fedorowicz A (2017). Coverage with Tetanus, Diphtheria, and Acellular Pertussis Vaccine and Influenza Vaccine Among Pregnant Women - Minnesota, March 2013-December 2014. Mmwr. Morb. Mortal. Wkly. Rep..

[78] Rai R, Singh DK (2012). Neonatal tetanus: a continuing challenge. Indian. J. Pediatr..

[79] Thwaites CL, Beeching NJ, Newton CR (2015). Maternal and neonatal tetanus. Lancet.

[80] Organization, W. H. In *Weekly epidemiological record*, Vol. 81 197-208 (2006).

[81] Blencowe H, Lawn J, Vandelaer J, Roper M, Cousens S (2010). Tetanus toxoid immunization to reduce mortality from neonatal tetanus. Int. J. Epidemiol..

[82] Hall CB (2001). Respiratory syncytial virus and parainfluenza virus. N. Engl. J. Med..

[83] Wheeler SM, Dotters-Katz S, Heine RP, Grotegut CA, Swamy GK (2015). Maternal Effects of Respiratory Syncytial Virus Infection during Pregnancy. Emerg. Infect. Dis..

[84] Chu HY (2016). Clinical Presentation and Birth Outcomes Associated with Respiratory Syncytial Virus Infection in Pregnancy. PLoS. One..

[85] Kwon YM (2014). Maternal antibodies by passive immunization with formalin inactivated respiratory syncytial virus confer protection without vaccine-enhanced disease. Antivir. Res..

[86] Blanco JCG (2017). Preclinical assessment of safety of maternal vaccination against respiratory syncytial virus (RSV) in cotton rats. Vaccine.

[87] Garg R (2016). Maternal immunization with respiratory syncytial virus fusion protein formulated with a novel combination adjuvant provides protection from RSV in newborn lambs. Vaccine.

[88] Glenn GM (2015). Modeling maternal fetal RSV F vaccine induced antibody transfer in guinea pigs. Vaccine.

[89] Cromer D, van Hoek AJ, Newall AT, Pollard AJ, Jit M (2017). Burden of paediatric respiratory syncytial virus disease and potential effect of different immunisation strategies: a modelling and cost-effectiveness analysis for England. Lancet Public Health.

[90] Anderson LJ (2013). Strategic priorities for respiratory syncytial virus (RSV) vaccine development. Vaccine.

[91] da Silva e Sa GR (2011). Pregnancy outcomes following rubella vaccination: a prospective study in the state of Rio de Janeiro, Brazil, 2001-2002. J. Infect. Dis..

[92] Suzano CE, Amaral E, Sato HK, Papaiordanou PM (2006). The effects of yellow fever immunization (17DD) inadvertently used in early pregnancy during a mass campaign in Brazil. Vaccine.

[93] Loubet P (2014). Should expectant mothers be vaccinated against flu? A safety review. Expert. Opin. Drug. Saf..

[94] Georgiades P, Ferguson-Smith AC, Burton GJ (2002). Comparative developmental anatomy of the murine and human definitive placentae. Placenta.

[95] Cox B (2009). Comparative systems biology of human and mouse as a tool to guide the modeling of human placental pathology. Mol. Syst. Biol..

[96] Grigsby PL (2016). Animal Models to Study Placental Development and Function throughout Normal and Dysfunctional Human Pregnancy. Semin. Reprod. Med..

[97] Zucker I (2017). Risk mitigation for children exposed to drugs during gestation: A critical role for animal preclinical behavioral testing. Neurosci. Biobehav. Rev..

[98] Nosten F (2006). Antimalarial drugs in pregnancy: a review. Curr. Drug. Saf..

[99] Clark RL (2009). Embryotoxicity of the artemisinin antimalarials and potential consequences for use in women in the first trimester. Reprod. Toxicol..

[100] Bialas KM (2015). Maternal CD4+T cells protect against severe congenital cytomegalovirus disease in a novel nonhuman primate model of placental cytomegalovirus transmission. Proc. Natl. Acad. Sci. USA.

[101] Choi KY, Root M, McGregor A (2016). A Novel Non-Replication-Competent Cytomegalovirus Capsid Mutant Vaccine Strategy Is Effective in Reducing Congenital Infection. J. Virol..

[102] Padilla-Carlin DJ, McMurray DN, Hickey AJ (2008). The guinea pig as a model of infectious diseases. Comp. Med..

[103] Ho EL, Lukehart SA (2011). Syphilis: using modern approaches to understand an old disease. J. Clin. Invest..

[104] Bakardjiev AI, Stacy BA, Fisher SJ, Portnoy DA (2004). Listeriosis in the pregnant guinea pig: a model of vertical transmission. Infect. Immun..

[105] Gitter M, Richardson C, Boughton E (1986). Experimental infection of pregnant ewes with Listeria monocytogenes. Vet. Rec..

[106] Smith MA (2003). Nonhuman primate model for Listeria monocytogenes-induced stillbirths. Infect. Immun..

[107] Lecuit M (2005). Understanding how Listeria monocytogenes targets and crosses host barriers. Clin. Microbiol. Infect..

[108] Disson O (2008). Conjugated action of two species-specific invasion proteins for fetoplacental listeriosis. Nature.

[109] Damman A (2015). Modelling the spread of bovine viral diarrhea virus (BVDV) in a beef cattle herd and its impact on herd productivity. Vet. Res..

[110] Newcomer BW, Walz PH, Givens MD, Wilson AE (2015). Efficacy of bovine viral diarrhea virus vaccination to prevent reproductive disease: a meta-analysis. Theriogenology.

[111] Duffy MR (2009). Zika virus outbreak on Yap Island, Federated States of Micronesia. N. Engl. J. Med..

[112] Musso D, Nilles EJ, Cao-Lormeau VM (2014). Rapid spread of emerging Zika virus in the Pacific area. Clin. Microbiol. Infect..

[113] Franca GV (2016). Congenital Zika virus syndrome in Brazil: a case series of the first 1501 livebirths with complete investigation. Lancet.

[114] Khrustalev VV, Khrustaleva TA, Sharma N, Giri R (2017). Mutational Pressure in Zika Virus: Local ADAR-Editing Areas Associated with Pauses in Translation and Replication. Front. Cell. Infect. Microbiol..

[115] Mansuy JM (2016). Zika virus in semen and spermatozoa. Lancet Infect. Dis..

[116] Govero J (2016). Zika virus infection damages the testes in mice. Nature.

[117] Vermillion MS (2017). Intrauterine Zika virus infection of pregnant immunocompetent mice models transplacental transmission and adverse perinatal outcomes. Nat. Commun..

[118] Quicke KM (2016). Zika Virus Infects Human Placental Macrophages. Cell. Host. Microbe.

[119] Tabata T (2016). Zika Virus Targets Different Primary Human Placental Cells, Suggesting Two Routes for Vertical Transmission. Cell. Host. Microbe.

[120] Atkinson B (2017). Presence and Persistence of Zika Virus RNA in Semen, United Kingdom, 2016. Emerg. Infect. Dis..

[121] Musso D (2015). Potential sexual transmission of Zika virus. Emerg. Infect. Dis..

[122] Bhatnagar J (2017). Zika Virus RNA Replication and Persistence in Brain and Placental Tissue. Emerg. Infect. Dis..

[123] Sapparapu G (2016). Neutralizing human antibodies prevent Zika virus replication and fetal disease in mice. Nature.

[124] Pardy RD (2017). Analysis of the T Cell Response to Zika Virus and Identification of a Novel CD8+T Cell Epitope in Immunocompetent Mice. PLoS. Pathog..

[125] World Health, O. WHO Vaccine Pipeline Tracker. (2017).

[126] Muthumani K, G. B, Agarwal S (2016). In vivo protection against ZIKV infection and pathogenesis through passive antibody transfer and active immunisation with a prMEnv DNAvaccine. Npj Vaccin..

[127] Richner JM (2017). Modified mRNA Vaccines Protect against Zika Virus Infection. Cell.

[128] Pardi N (2017). Zika virus protection by a single low-dose nucleoside-modified mRNA vaccination. Nature.

[129] Plotkin SA, Liese J, Madhi SA, Ortiz E (2011). A DTaP-IPV//PRP approximately T vaccine (Pentaxim): a review of 16 years’ clinical experience. Expert. Rev. Vaccin..

[130] Moyer TJ, Zmolek AC, Irvine DJ (2016). Beyond antigens and adjuvants: formulating future vaccines. J. Clin. Invest..

[131] Gomes, A. C., Mohsen, M. & Bachmann, M. F. Harnessing Nanoparticles for Immunomodulation and Vaccines. *Vaccines* **5**, 6 (2017). 10.3390/vaccines5010006 PMC5371742 28216554

[132] Garcia-Sastre A, Mena I (2013). Novel vaccine strategies against emerging viruses. Curr. Opin. Virol..

[133] Jones GJ, Boles C, Roper RL (2014). Raccoonpoxvirus safety in immunocompromised and pregnant mouse models. Vaccine.

[134] Dudek T, Knipe DM (2006). Replication-defective viruses as vaccines and vaccine vectors. Virology.

[135] Nogales A, Martínez-Sobrido L (2017). Reverse Genetics Approaches for the Development of Influenza Vaccines. Int. Journal. of Mol. Sci..

[136] Baker SF (2013). Protection against lethal influenza with a viral mimic. J. Virol..

[137] Katsura H (2012). A replication-incompetent virus possessing an uncleavable hemagglutinin as an influenza vaccine. Vaccine.

[138] Powell TJ, Silk JD, Sharps J, Fodor E, Townsend AR (2012). Pseudotyped influenza A virus as a vaccine for the induction of heterotypic immunity. J. Virol..

[139] Shinya K (2004). PB2 amino acid at position 627 affects replicative efficiency, but not cell tropism, of Hong Kong H5N1 influenza A viruses in mice. Virology.

[140] Uraki R (2013). A novel bivalent vaccine based on a PB2-knockout influenza virus protects mice from pandemic H1N1 and highly pathogenic H5N1 virus challenges. J. Virol..

[141] Victor ST, Watanabe S, Katsura H, Ozawa M, Kawaoka Y (2012). A replication-incompetent PB2-knockout influenza A virus vaccine vector. J. Virol..

[142] Masic A, Pyo HM, Babiuk S, Zhou Y (2013). An eight-segment swine influenza virus harboring H1 and H3 hemagglutinins is attenuated and protective against H1N1 and H3N2 subtypes in pigs. J. Virol..

[143] Eberhardt CS (2017). Pertussis Antibody Transfer to Preterm Neonates After Second- Versus Third-Trimester Maternal Immunization. Clin. Infect. Dis..

[144] Adachi K, Nielsen-Saines K, Klausner JD (2016). Chlamydia trachomatis Infection in Pregnancy: The Global Challenge of Preventing Adverse Pregnancy and Infant Outcomes in Sub-Saharan Africa and Asia. Biomed. Res. Int..

[145] Ledger WJ (2008). Perinatal infections and fetal/neonatal brain injury. Curr. Opin. Obstet. Gynecol..

[146] Schrag S, Gorwitz R, Fultz-Butts K, Schuchat A (2002). Prevention of perinatal group B streptococcal disease. Revised guidelines from CDC. MMWR Recomm. Rep..

[147] Arora N, Sadovsky Y, Dermody TS, Coyne CB (2017). Microbial Vertical Transmission during Human Pregnancy. Cell. Host Microbe.

[148] Lago EG (2016). Current Perspectives on Prevention of Mother-to-Child Transmission of Syphilis. Cureus.

[149] De Santis M (2012). Syphilis Infection during pregnancy: fetal risks and clinical management. Infect. Dis. Obstet. Gynecol..

[150] Natama HM (2017). Diagnosing congenital malaria in a high-transmission setting: clinical relevance and usefulness of P. falciparum HRP2-based testing. Sci. Rep..

[151] White NJ (2014). Malaria. Lancet.

[152] Kovacs SD, Rijken MJ, Stergachis A (2015). Treating severe malaria in pregnancy: a review of the evidence. Drug. Saf..

[153] Wong SY, Remington JS (1994). Toxoplasmosis in pregnancy. Clin. Infect. Dis..

[154] Rodrigues IM (2014). Assessment of laboratory methods used in the diagnosis of congenital toxoplasmosis after maternal treatment with spiramycin in pregnancy. BMC Infect. Dis..

[155] Cheeran MC, Lokensgard JR, Schleiss MR (2009). Neuropathogenesis of congenital cytomegalovirus infection: disease mechanisms and prospects for intervention. Clin. Microbiol. Rev..

[156] Pass RF, Fowler KB, Boppana SB, Britt WJ, Stagno S (2006). Congenital cytomegalovirus infection following first trimester maternal infection: symptoms at birth and outcome. J. Clin. Virol..

[157] Planitzer CB, Saemann MD, Gajek H, Farcet MR, Kreil TR (2011). Cytomegalovirus neutralization by hyperimmune and standard intravenous immunoglobulin preparations. Transplantation.

[158] Jamieson DJ, Uyeki TM, Callaghan WM, Meaney-Delman D, Rasmussen SA (2014). What obstetrician-gynecologists should know about Ebola: a perspective from the Centers for Disease Control and Prevention. Obstet. Gynecol..

[159] Black BO, Caluwaerts S, Achar J (2015). Ebola viral disease and pregnancy. Obstet. Med..

[160] Nelson JM, Griese SE, Goodman AB, Peacock G (2016). Live neonates born to mothers with Ebola virus disease: a review of the literature. J. Perinatol..

[161] Avgil M, Ornoy A (2006). Herpes simplex virus and Epstein-Barr virus infections in pregnancy: consequences of neonatal or intrauterine infection. Reprod. Toxicol..

[162] McAllister SC, Schleiss MR (2014). Prospects and perspectives for development of a vaccine against herpes simplex virus infections. Expert. Rev. Vaccin..

[163] Drake AL, Wagner A, Richardson B, John-Stewart G (2014). Incident HIV during pregnancy and postpartum and risk of mother-to-child HIV transmission: a systematic review and meta-analysis. PLoS. Med..

[164] Tobin NH, Aldrovandi GM (2013). Immunology of pediatric HIV infection. Immunol. Rev..

[165] Lamont RF (2011). Parvovirus B19 infection in human pregnancy. BJOG.

[166] Miller E, Cradock-Watson JE, Pollock TM (1982). Consequences of confirmed maternal rubella at successive stages of pregnancy. Lancet.

[167] Li AM, Ng PC (2005). Severe acute respiratory syndrome (SARS) in neonates and children. Arch. Dis. Child. Fetal Neonatal Ed..

[168] Stockman LJ, Lowther SA, Coy K, Saw J, Parashar UD (2004). SARS during pregnancy, United States. Emerg. Infect. Dis..

[169] De Paschale M, Clerici P (2016). Microbiology laboratory and the management of mother-child varicella-zoster virus infection. World J. Virol..

[170] Miranda-Filho Dde B (2016). Initial Description of the Presumed Congenital Zika Syndrome. Am. J. Public Health.

